# Robust and Compact Electrostatic Comb Drive Arrays for High-Performance Monolithic Silicon Photonics

**DOI:** 10.3390/mi16101102

**Published:** 2025-09-28

**Authors:** Mohammadreza Fasihanifard, Muthukumaran Packirisamy

**Affiliations:** Optical-Bio Microsystems Laboratory, Department of Mechanical, Industrial and Aerospace Engineering, Concordia University, 1515 St. Catherine W., Montreal, QC H3G 2W1, Canada

**Keywords:** electrostatic comb drive, silicon photonics, MEMS actuators, force intensity optimization, slab waveguide actuation

## Abstract

Actuating monolithic photonic components (particularly slab waveguides) requires higher force due to their inherent stiffness. However, two primary constraints must be addressed: actuator footprint and fabrication limits. Increasing the number of fingers to provide the required force is not a viable solution due to space constraints, and we must also adhere to the process design kits of standard fabrications and respect their design limits. Therefore, it is crucial to increase the actuator force output without significantly enlarging the actuator footprint while maintaining the necessary travel range. In order to achieve this, we utilize arrays of electrostatic comb drives, with each repeating cell geometry optimized to produce the highest force per actuator footprint. Our optimization strategy focuses on finger geometry, the arrangement of fingers and arms design in the comb structure, including the number of fingers per arm and arm length, ensuring that each repeating cell delivers maximum force per unit area or force intensity. Co-optimizing a repeatable, footprint-optimized comb-array unit cell (arm length, arm width, finger pitch, finger count) and validating it against an asymmetric slab waveguide load, we reach a maximum pre-pull-in force intensity of about 342 N m^−2^ at 70 V with about 6 µm travel, confirmed by analytical modeling, numerical simulation, and measurement. Despite fabrication challenges such as over-etching and variations in electrode dimensions, detailed SEM analyses and correction functions ensure that the theoretical models closely match the experimental data, confirming the robustness and accuracy of the design. These optimized actuators, capable of achieving substantial force output without sacrificing travel range or mechanical stability, are particularly effective for applications in optical beam steering for in-plane silicon-photonics and related optical microsystems applications.

## 1. Introduction

### 1.1. Background

Active and tunable silicon photonic components have garnered significant attention over the past few decades due to their potential to revolutionize optical communication and sensor systems. Integrating photonic devices with optical microsystems allows for reconfigurable photonic circuits capable of addressing multiple functions simultaneously for various applications, such as optical network components and sensors [1,2,3,4,5,6]. Mechanical tuning of photonic devices is particularly advantageous because of its compatibility with existing fabrication processes [1,7,8] and provides a wider dynamic range.

However, conventional electrostatic actuators face limitations such as non-linear deflection, limited displacement, low force, and high actuation voltage [9]. To overcome these challenges, previous research introduced electrostatic comb drives with triangular-shaped finger configurations, offering an optimized balance between force intensity and traveling range [10,11,12,13,14,15,16]. These designs demonstrated potential for enhancing the performance of active silicon photonic devices that require high force and extensive travel range [8,17,18,19].

Building on these advancements, this paper presents a novel approach to actuate a continuous, stiff slab waveguide using optimized electrostatic comb actuators. This study eliminates the arm length restriction and fully optimizes both the arm size and finger geometry to improve actuator performance. The design is specifically optimized for force intensity (force per footprint of the actuator) and traveling range, which are critical parameters for effective actuation in silicon photonics. Providing high force with a low footprint for the required travel range paves the way for devices with continuous waveguide actuation that have high mechanical stiffness. This approach offers significant benefits, including lower loss, reduced polarization extinction ratio (PER), and improved overall performance in active photonic systems [20,21].

In optical MEMS devices, segmented designs refer to configurations where there are deliberate separations or gaps between components, such as in the case of switches where the light path is interrupted. These gaps can lead to optical losses due to diffraction, scattering, and reflection. Conversely, monolithic designs feature continuous waveguides without such interruptions, leading to seamless optical paths that reduce these losses. The distinction between these designs is critical for optimizing the performance of photonic devices. Table 1 summarizes the key features and differences between monolithic and segmented devices in silicon photonics, focusing on aspects such as optical coupling, fabrication, actuation, tuning methods, and applications.

In terms of optical coupling and alignment, monolithic devices offer significant advantages. They do not suffer from coupling challenges and exhibit low insertion loss due to the absence of diffraction and scattering at gaps. This continuous and predictable change in optical properties is crucial for high optical efficiency. Segmented devices, however, face coupling challenges, needing to manage the air gap after alignment. This can lead to potential diffraction, scattering, or loss, though they are capable of large displacements for significant optical path changes [17,20,22,23,24,25,26].

From a fabrication standpoint, monolithic devices are simpler to manufacture. They avoid the need for special mechanical controls and design considerations required to maintain coupling efficiency. This simplicity is due to the continuous waveguides used. In contrast, segmented devices face challenges in fabricating robust structures that can maintain coupling efficiency and stability over time, which increases complexity [5,6,24,25,27].

**Table 1:** Comparison of monolithic and segmented devices in silicon photonics Summary of features and differences between monolithic and segmented devices in silicon photonics, focusing on aspects such as optical coupling, fabrication, actuation, tuning methods, and applications.

Feature	Monolithic Devices			Segmented Devices	Refs.
Optical coupling and alignment	No coupling challenges. Continuous, predictable changes in optical properties. Low insertion loss due to the absence of diffraction and scattering at gaps. Challenge for large displacements.			Coupling challenges; needs to manage the air gap after alignment. Prone to diffraction, scattering, or loss depending on gap size. Capable of large displacements for significant optical path changes.	[15,17,20,22,23,24,25]
Fabrication	Simpler fabrication due to continuous waveguides, Avoiding special mechanical controls and design considerations.			Challenges in fabricating robust structures to increase coupling efficiency stability and avoid drift over time.	[5,6,24,25,27]
Actuation	Smaller actuation range due to material and design constraints. A larger actuator is required due to high stiffness.			A larger actuation range is achievable. Smaller actuators.	[17,20,22,23,24,25,28,29,30]
Tuning Method and Applications	Waveguide Routing Control	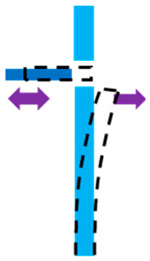	Mechanical Modification of Waveguide Routing: Optical Switches Wavelength Switches: Optical Signal Modulators	Coupling/Decoupling Light Between Waveguides: Optical Switches Wavelength Switches: Optical attenuators Moving mirrors Tunable VCSELs.	[5,8,15,17,20,22,24,26,30,31,32,33,34,35,36,37,38,39,40]
	Evanescent Field Interactions	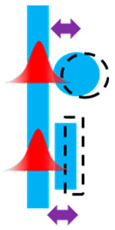	Intensity Modulators Optical Switches Tunable Filters Tunable Resonators Sensors.	Evanescent tuning is typically used for monolithic devices.	[1,15,22,30,31,41,42,43]
	Waveguide Property Modification	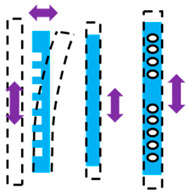	Modifying Waveguide Properties (Strain/Stress/Deforming/Shortening Lengthening): Tunable filters Optical modulators wavelength converter Deformable gratings Moving waveguides.	Waveguide Property Modification is typically used for monolithic devices.	[19,31,33,44,45,46,47,48]

Regarding actuation, monolithic devices require larger actuators due to the high stiffness of continuous waveguides, resulting in a smaller actuation range due to material and design constraints. Segmented devices, however, achieve larger actuation ranges with smaller actuators. This makes them suitable for applications that require significant optical path changes [17,20,22,23,24,25,28,29,30].

The three main mechanical tuning methods for photonic devices are waveguide routing control, evanescent field interactions, and waveguide property modification. For waveguide routing control, monolithic devices excel due to their continuous optical paths, which facilitate applications such as optical switches, wavelength switches, and optical signal modulators. These applications benefit from the predictable changes in optical properties provided by monolithic designs. Segmented devices are also used for waveguide routing control, where they enable coupling and decoupling of light between waveguides, suitable for optical switches, wavelength switches, optical attenuators, moving mirrors, and tunable Vertical-Cavity Surface-Emitting Lasers (VCSELs) [5,8,17,20,22,24,30,31,32,33,34,35,36,37,38,39,40,47].

In evanescent field interactions, monolithic devices are highly effective because they maintain continuous waveguides that allow for consistent evanescent field interactions. This makes them suitable for intensity modulators, optical switches, tunable filters, and resonators. Segmented devices, while less commonly used for evanescent tuning, can still be applied in scenarios where precise control of light coupling is needed [1,22,30,31,41,42,43].

For waveguide property modification, monolithic devices are particularly advantageous due to their ability to modify waveguide properties such as strain, stress, deformation, shortening, or lengthening. This enables applications in tunable filters, optical modulators, wavelength converters, and deformable gratings. Segmented devices are less commonly used for such modifications but can still find applications in stress-optic devices and other scenarios where the physical properties of the waveguide need to be adjusted [30,32,44,45,46].

Understanding these distinctions underscores the significance of optimizing comb actuators specifically for monolithic waveguide actuation. The proposed design in this study aims to maximize force intensity and travel range while ensuring mechanical reliability, addressing the unique needs of monolithic device operation. The study validates the proposed design through detailed simulations and experimental studies, demonstrating its effectiveness in achieving the desired mechanical and optical performance. This research aims to contribute to the development of tunable photonic devices by providing a robust and efficient actuation mechanism suitable for various applications in silicon photonics.

### 1.2. Monolithic Device Actuation: The Need for High Force and Large Traveling Range

Actuating monolithic devices, which involve continuous waveguides without gaps or discontinuities, presents unique challenges that require careful optimization of electrostatic comb-drive designs. In contrast to devices with gaps, where actuation primarily focuses on achieving sufficient displacement to open or close the gap, continuous waveguide actuation demands both high force intensity and large travel range due to the waveguide’s mechanical rigidity. Monolithic waveguides, particularly those fabricated from stiff materials like silicon, exhibit high mechanical rigidity. Accordingly, actuators must generate substantial force per unit area (force intensity) to overcome this stiffness and enable precise, reliable actuation.

Large displacement is essential for expanding the tuning range of photonic devices. A larger travel range allows modifications to the waveguide geometry (bending, stretching, or compressing) that can significantly alter its optical properties and enable highly tunable devices.

Monolithic devices with continuous waveguide actuation, without gaps, offer several clear benefits over traditional gap-based actuation methods:

- Lower Loss: The absence of gaps eliminates optical losses due to diffraction, scattering, and reflections, ensuring higher optical efficiency and improved signal transmission.
- Reduced Polarization Extinction Ratio (PER): Continuous waveguides maintain a more consistent polarization state, leading to lower PER and enhanced polarization stability, which is critical for many photonic applications.
- Improved Performance: The combination of lower loss, reduced PER, and the ability to achieve mechanically and optically robust structure translates to significantly improved performance and broader functionality for tunable photonic devices.

### 1.3. System Overview

Optical microsystems technology offers a promising approach for miniaturizing devices and integrating them with silicon photonics, enabling the development of Reconfigurable Photonic Integrated Circuits (PICs). These circuits provide robust and low-power platforms for tunable photonic devices such as phase shifters, couplers, switches, and resonators [6,41]. The main actuation methods for microsystems are electrostatic, electromagnetic, piezoelectric, and electrothermal mechanisms [48]. Among these, electrostatic actuation stands out for its low power consumption, high speed, and ease of control, making it highly compatible with silicon photonics [22,31,32,49].

We use an asymmetric slab waveguide fabricated using a silicon-on-insulator (SOI) process. While not optically optimized here, it serves as a representative platform for demonstrating the functionality and effectiveness of the micro electromechanical actuator design. Additionally, it has potential applications in creating mechanically active beam steering devices in silicon photonics [19].

The primary focus of this research is to optimize electrostatic comb drive actuators for actuating continuous and stiff slab waveguides. The asymmetric slab waveguide provides a representative sample to validate the mechanical performance of the actuator. Although optical optimization is beyond the scope of this paper, the slab waveguide’s potential for future photonic applications justifies its use. Specifically, it can be employed as a platform for fabricating additional waveguides, enabling the creation of complex photonic structures. In future studies, this waveguide could be optimized for forming the external cavity of a tunable laser, among other applications. Therefore, the current study lays the groundwork for future developments by ensuring effective interaction between the MEMS actuator and such a platform

In the subsequent sections, we will explore the design methodology and optimization strategies required to achieve the necessary performance metrics for monolithic device actuation, focusing on high force intensity and large traveling range. We will also demonstrate the practicality of this design by developing a MEMS platform that can support various types of waveguides, specifically emphasizing a stiff asymmetric slab waveguide. Detailed simulations and experimental results will be presented to validate the robustness and efficiency of the proposed approach in meeting these critical performance requirements.

**Figure 1:**
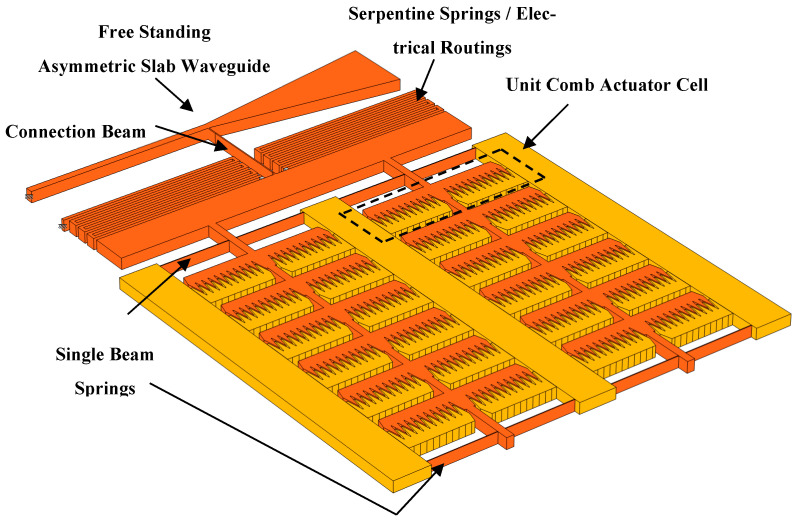
Schematic of the actuation setup for the stiff monolithic slab waveguide on an SOI MEMS platform. The diagram shows the free-standing asymmetric slab waveguide, connection beam, single beam springs, electrical routings, and unit comb actuator cells. This optimized design ensures high force intensity and mechanical stability, essential for precise actuation in silicon photonics applications.

In this study, we developed an SOI platform that supports the integration of waveguides on top. Figure 1 shows the diagram below illustrates the key components and layout of the actuation setup, highlighting the free-standing asymmetric slab waveguide, connection beams, single beam springs, and unit comb actuator cells. This configuration ensures high force intensity and a compact footprint, which are critical for effective actuation in silicon photonics. While the same design procedure can be applied for SOI waveguides with adjusted dimensions, this paper focuses on an SOI MEMS platform intended for subsequent integration of SiN waveguides.

### 1.4. Array-Level Optimization for Footprint and Force

This study builds on our earlier angled-finger actuator work [16]. The overlap is the same basic concept of triangular fingers and electrostatic actuation. What is new here is that we move from single-comb tuning to array-level optimization with a repeatable, footprint-optimized unit cell that can be tiled to reach a target force while keeping the travel and stability. We co-optimize arm length, arm width, finger pitch, and the number of fingers per arm, so the whole comb array is optimized, not just the finger.

We also replace the simple spring load used before with a more realistic monolithic photonic component, an asymmetric slab waveguide, so the measured force, stiffness, and travel reflect a real device load. With this array and arm optimization, the measured maximum force intensity, before pull-in, improves from about 200 N m^−2^ at 100 V with about 3.5 µm travel in the earlier design to about 342 N m^−2^ at 70 V with about 6 µm travel. The higher performance at lower voltage comes from the array layout and the arm co-design.

## 2. Materials and Methods

### 2.1. Analytical and Parametric Study

Initially, an analytical model was developed to understand the fundamental dynamics of the electrostatic comb drive actuators. This model provided insights into the relationships between key parameters such as force intensity, travel range, and mechanical stability. A comprehensive parametric study was conducted to identify the optimal configurations for the comb drive actuators. The study examined key parameters including finger geometry [15,16], the number of fingers per arm, arm length and width, and the overall actuator footprint. This parametric study facilitated the selection of designs that provided a balance between high force intensity, adequate travel range, and mechanical stability.

### 2.2. Numerical Simulation

Following the analytical and parametric studies, numerical simulations were performed to validate and refine the selected designs. These simulations were conducted using COMSOL Multiphysics version 6.0, focusing on force intensity, travel range, and mechanical stability. Finite Element Analysis (FEA) was utilized to assess the performance of various configurations, providing detailed insights into the mechanical and electrostatic behavior of the comb drive actuators.

### 2.3. Fabrication Process

In this work we fabricated and tested only the MEMS electrostatic comb actuators on a thick silicon-on-insulator (SOI) platform. No optical waveguides were fabricated on top in this fabrication run. We include below a proposed integration flow for adding silicon nitride waveguides on the same thick SOI platform in a future iteration, and we discuss process compatibility and expected risks. All measurements and SEMs reported in this paper are from the MEMS-only devices.

**Figure 2:**
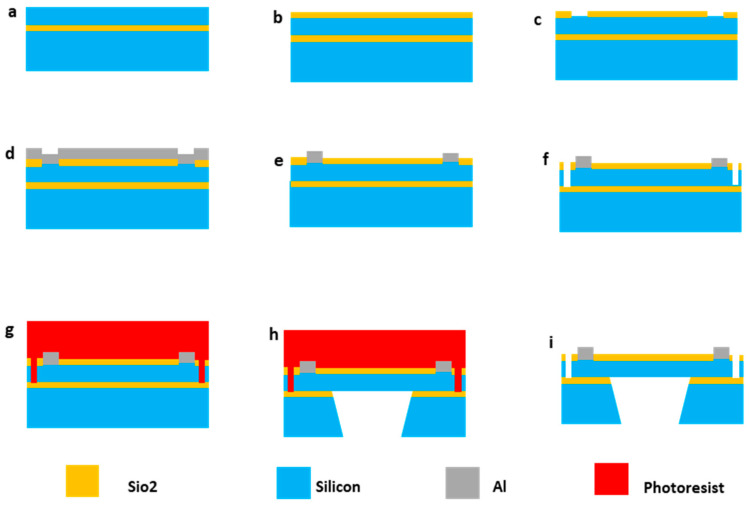
Diagram of the MEMS fabrication process used in this work. The diagram provides a step-by-step visual representation of the fabrication process. It starts with an SOI wafer (**a**) and includes stages such as oxide growth and isolation etching (**b**), oxide patterning (**c**), pad metal deposition (**d**), and the liftoff process (**e**). It then details deep silicon etching (**f**), application of a protective layer (**g**), etching of the backside substrate and removal of buried oxide (BOX) (**h**), and concludes with the removal of the protective layer (**i**) to expose the finished comb drive structures, which are then ready for dicing and testing.

The fabrication of the electrostatic comb actuators was executed using the PiezoMUMPs [50] process at the MEMSCAP foundry, specifically tailored for Silicon-on-Insulator (SOI) substrates. The process is initiated with an SOI wafer comprising a 10 µm silicon layer, a 1 µm oxide layer, and a 400 µm substrate. The fabrication commenced with the doping and annealing of the silicon layer. This was followed by the removal of the phosphosilicate glass (PSG) layer through wet chemical etching. Subsequent steps included the patterning and etching of the silicon down to the oxide layer to form mechanical and electrical structures. An insulating thermal oxide layer of 2000 Å was grown, patterned, and etched using reactive ion etching (RIE) (Figure 2).

Process compatibility: All geometries were laid out to comply with the PiezoMUMPs design rules. We used the 10 µm Silicon device and set all critical dimensions like minimum feature size, spacing, and electrode gaps, especially for angled tips (≥2 µm, and ≥3 µm for non-orthogonal/angled features) at or above the PDK minima. Required layer clearances were observed, and release/anchor layouts were sized for reliable under-etch and were budgeted with a few-micrometer margin. Release and anchor layouts were sized for reliable under-etch, with anchors set back from trench edges, stator segments kept ≈ 200 µm to avoid deep-trench under-etch issues and long released beams were kept sufficiently wide to avoid excessive bending after release. The fabrication limits listed in Table 2 (e.g., layer thicknesses, minimum gaps, arm widths/pitches) were chosen directly from these rules and marked as fabrication process constraints (FPC).

### 2.4. Data Analysis and Correction

Spring over-etching was addressed by analyzing SEM images to determine actual fabricated dimensions and adjusting finite element models (FEM) to calculate corrected stiffness values. Electrode dimension adjustments were made by applying correction functions to account for widened finger angles and electrode gap discrepancies. Adjusted simulations ensured alignment between theoretical models and experimental data.

### 2.5. Experimental Validation

The experimental validation of the optimized electrostatic comb drive actuators was carried out through a series of tests to compare their performance with the simulation results. The fabricated devices were meticulously characterized using a high-resolution imaging system and a precision micromechanical testing setup.

#### Measurement Setup

**Figure 3:**
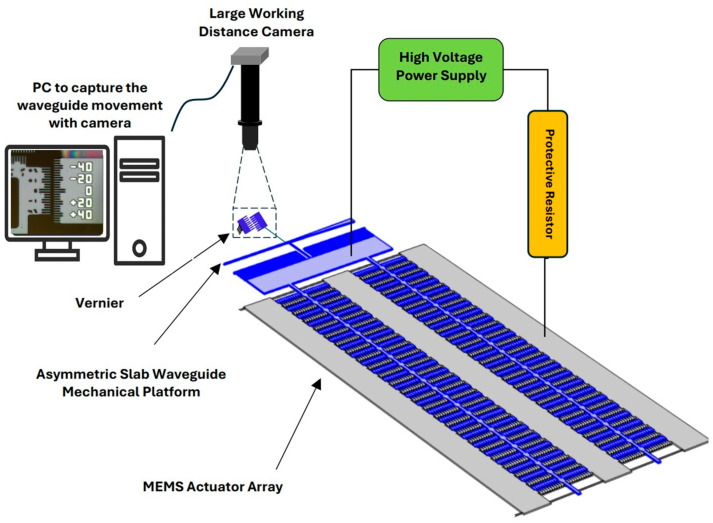
The diagram depicts the experimental arrangement for testing the MEMS actuator. A DC bias is supplied to the electrodes by a high-voltage power source, and a 100 kΩ protective resistor is included in the circuit to mitigate overcurrent risks during pull-in scenarios. The setup incorporates a high-magnification zoom system with a substantial working distance to track electrode movements in real-time, accompanied by a vernier system for precise measurement of displacement.

Figure 3 illustrates the detailed schematic of the testing arrangement. A high-voltage power supply is connected to the MEMS actuator via a protective resistor, ensuring safe and accurate testing. The device features an asymmetric slab waveguide integrated with a vernier fabricated from the same silicon layer, enabling precise measurement of movement at the middle of the slab waveguide, representing the MEMS actuator displacement, with an accuracy of 0.25 μm. A camera system monitors and measures the waveguide’s movement, with the data controlled and recorded by a computer system.

The FemtoTools FT-MTA02 Micromechanical Testing and Assembly Station was essential for our experimental setup, offering precise handling and high-resolution imaging capabilities. The FT-MTA02 Initially equipped with a 3-megapixel CMOS USB camera and an adjustable zoom system, it provided a field of view from 15 mm × 11 mm to 2.2 mm × 1.7 mm, with an optical resolution of 3 µm. To achieve the submicron precision necessary for our measurements, we enhanced the imaging system by installing a 20× objective lens, enabling us to detect displacements as small as 0.2 µm. This upgrade, combined with the vernier scale, allowed us to measure movements with an accuracy of 0.25 µm.

The FT-MTA02’s robotic arm and micropositioner were critical for testing unpackaged dies with high accuracy. We used a 20 µm needle to probe the device pads, ensuring precise voltage application. Each device underwent triple testing to ensure data consistency and reliability, which was reflected in the error bars in our graphs. The electrostatic force at various voltages was determined from the measured displacement data, utilizing the known stiffness of the comb drives to evaluate actuator performance accurately. The force intensity for each design was calculated based on the force and the measured footprint area of the comb drive, providing a comprehensive assessment of actuator efficiency. 

## 3. Results

In previous studies, the focus was primarily on optimizing finger geometry, while the overall comb configuration received less attention. However, in applications requiring higher force intensity, utilizing an array of comb actuators becomes necessary. This section discusses the process of determining the optimum arm length and finger geometry at the same time when considering arrays of actuators to achieve the required force intensity while maintaining mechanical stability and avoiding pull-in.

The design challenge involves finding the balance between maximizing force intensity and ensuring lateral stability. Each actuator array must include stators of sufficient width to maintain structural integrity. By optimizing the arm length, we ensure that the array configuration provides the highest force intensity achievable within the mechanical constraints. The width of each stator and the arm width are critical factors in preventing lateral instability and pull-in.

To determine the optimal arm length for arrays of comb actuators, we conducted simulations to evaluate the mechanical behavior and force output of various configurations. These simulations considered the necessary spacing for stators within the cell footprint and validated arm widths for stability.

Our approach involves adjusting the arm length and finger geometry to find the maximum force intensity for a given array configuration. By repeating the arrays with the optimized arm length, we can achieve the desired force intensity for different applications. This method allows for scalable adjustments to force intensity by varying the number of repeated arrays, ensuring that the actuator cell size does not adversely affect force intensity or increase the footprint inefficiently, thus providing flexibility in designing actuators for specific requirements. 

**Figure 4:**
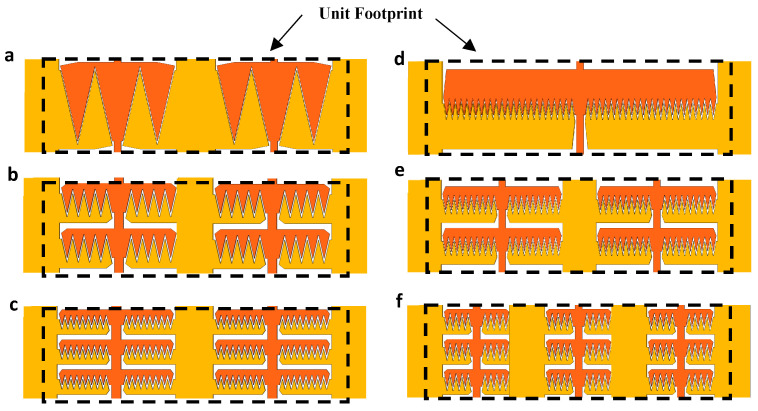
Array configurations and arm length variations. (**a**–**c**): Illustrate the composition of comb drives with varying finger pitches, showing the configuration of the combs and their number of stators and moving arms. (**d**–**f**): Show arrays with the same finger pitch but different arm lengths, highlighting how these design parameters determine the overall configuration of the combs. All six configurations are considered within a unit footprint to demonstrate how the combs can be embedded within that space.

As illustrated in Figure 4a–c, we studied various comb drive configurations with different numbers of fingers per arm to determine their influence on force intensity and actuator performance. Additionally, Figure 4d–f, presents arrays of a specific finger design with varying arm lengths, highlighting the optimization process to achieve maximum force intensity while ensuring mechanical stability and preventing pull-in. Through this analysis, we identified the optimal arm length that balances high force intensity with structural integrity, enabling scalable adjustments by repeating the optimized arrays.

Optimizing MEMS actuators using triangular fingers is effective due to their high force intensity. By conceptualizing each triangular (TRI) finger’s flank as an inclined segment of a parallel plate system, we analyze the electrostatic forces. The lateral forces cancel each other, leaving the vertical components to drive the actuator.

(1) $$F=\frac{\varepsilon_{0}\varepsilon_{r}hbV^{2}}{2c^{2}}\;\;$$

where $\epsilon_{0}$, and $\epsilon_{r}$ are vacuum and relative permittivity, h is the electrode thickness, V is the applied voltage, c is the electrode gap, and b is the effective electrode overlap. The gap c and overlap b are defined as:

(2) $$c=d\sin\left(\alpha \right)\;\;$$

(3) $$b=\frac{L_{f}}{2\sin\alpha }-d\cos\alpha \;\;\;$$

**Figure 5:**
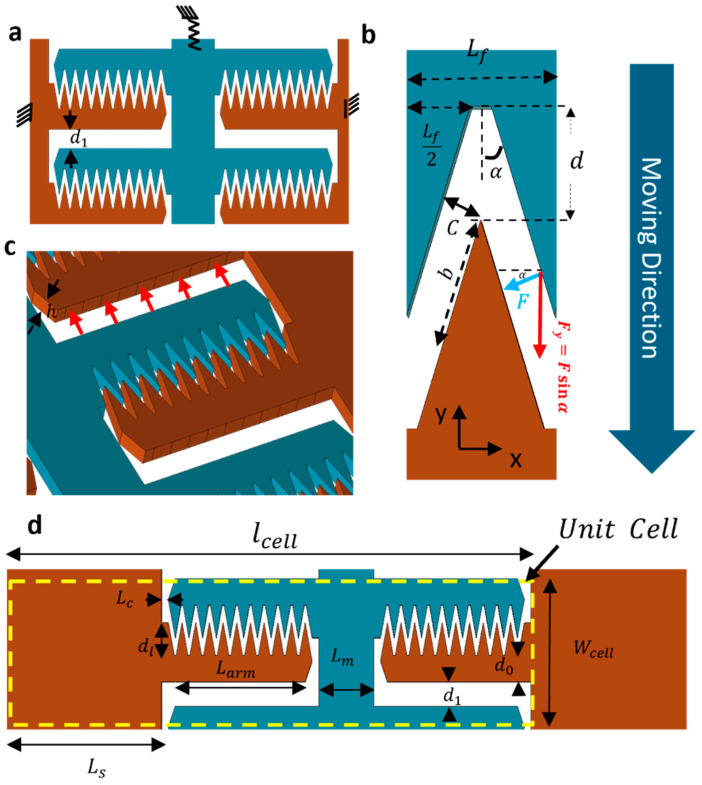
Dimensions of triangular finger comb drive. (**a**): Illustration of two unit cells of a comb drive with triangular fingers. (**b**): Detailed dimensions of a single triangular finger: $L_{f}$ (finger pitch), d (finger tip-to-tip separation), b (electrode overlap), α (half-angle of the finger tip), and c (gap between electrodes); the net vertical driving force $F_{y}$ component is indicated. (**c**): 3D view of a pair of arms with red arrows indicating the electrostatic forces between the moving arm and adjacent fixed arms, which act as parallel-plate electrostatic forces and are included in the total-force calculation. Also, it shows *h* (SOI silicon layer thickness). (**d**): Diagram of a unit cell, illustrating the lengths and widths of various components, including $L_{cell}$(cell length), $L_{s}$ (stator length), $L_{c}$ (connection beam length), $L_{arm}$ (arm length), $L_{m}$ (moving rail length), $W_{cell}$ (cell width), $d_{0}$ (arm width), and $d_{1}$ (distance to the next pair of electrodes). This figure focuses on the dimensions of the comb drive.

Finally, by incorporating these parameters into the force equation and combining the vertical components of the electrostatic forces acting on each finger, we determine the total driving force, $F_{y}$ (Figure 5):

(4) $$F_{y}=2\;F\sin\alpha$$

(5) $$F_{y}=\frac{\varepsilon_{0}\varepsilon_{r}hL_{f}V^{2}\;}{2d^{2}}\left(\;\frac{1}{\sin^{2}\alpha }-\frac{2d\cot\alpha }{L_{f}}\right)$$

The triangular finger configuration in MEMS actuators features an electrostatic force with two key components: the intrinsic electrostatic force seen in parallel plate setups and an enhancement factor due to triangular geometry. This enhancement factor grows as the electrode angle decreases, improving actuator efficiency [16]. The triangular design reduces gaps between electrodes, increasing electrostatic force over certain distances. Optimizing the angle α and finger dimensions allows precise control of the actuator’s travel range. This is essential for maintaining minimal gaps and achieving desired performance. Adjustments in these parameters ensure the optimal functionality of the actuator.

Additionally, it is crucial to consider the parallel plate capacitance created between adjacent cells, which impacts the overall electrostatic force calculation. This force acts in the reverse direction of the comb movement. The dimensions of this parallel plate are the length of the arm ($L_{arm}$) and the height of the arm (h), with the gap ($d_{1})$ between the adjacent cells. The electrostatic force ($F_{adj}$) for this configuration is given by:

(6) $$F_{adj}=\frac{\varepsilon_{0}\varepsilon_{r}hL_{f}V^{2}}{2{d_{1}}^{2}}\;\;$$

Thus, the pure force causing movement ($F_{y}$) would be the force derived in Equation (5) minus the opposing force $F_{adj}$. This ensures a more accurate representation of the force dynamics within the actuator system. As highlighted by the red arrows in Figure 5c.

### 3.1. Analytical Calculations and Numerical Simulations

#### 3.1.1. Design Configuration of Electrostatic Actuator Cell with Triangular Fingers

The design of the electrostatic actuator cell with triangular fingers involves several critical parameters that influence its performance and functionality. These parameters can be categorized into different types based on their nature and constraints (Table 2)

Some parameters are restricted by the fabrication process. For example, the element thickness (h) is determined by the SOI silicon layer thickness, which is a fixed constraint. Similarly, the finger separation (c) and the triangular finger angle (α) are limited by the precision of the manufacturing process and the risk of losing sharp definition during etching. This fabrication process constraints ensure that the design adheres to the capabilities and limitations of the available technology. In this study, the constrains were set based on the design rules of MEMSCAP [50].

Dependent parameters, such as the arm length ($L_{arm}$), cell length ($L_{cell}$), and cell width ($W_{cell}$), are calculated based on other values. These parameters define the physical geometry of the actuator and directly impact its force generation capabilities and mechanical behavior. They emerge as a result of the chosen design variables and fixed constraints.

Design restrictions are applied to ensure the structural integrity and operational stability of the actuator. Parameters such as the arm width ($d_{0}$), the distance to the next pair of electrodes ($d_{1}\;$), the moving rail length ($L_{m}$), and the maximum bending deflection ($D_{Max}$) fall into this category. These restrictions are crucial for preventing pull-in effects, lateral instability, and ensuring reliable performance under operational conditions.

The structure of the comb frame was selected to leverage specific mechanical properties essential for the actuator’s performance. Single beam springs are employed due to their selective stiffness characteristics, providing much higher stiffness along the direction parallel to the moving axis compared to the moving direction. This design minimizes lateral movements, which is crucial for the triangular finger configuration where the gap is particularly small, especially near the end of the actuator’s travel range. Unlike rectangular fingers, triangular fingers experience both gap-closing and area overlap situations, making lateral stability even more critical. Additionally, serpentine springs are designed to have minimal stiffness while being sufficiently wide to accommodate metal routing on top, adhering to the process design rules of MEMSCAP [50]. This combination of selective stiffness and appropriate spring design ensures the mechanical reliability and precision of the actuator. Optimization parameters are those that can be fine-tuned to enhance the performance of the actuator. For instance, the finger pitch ($L_{f}$) and the number of fingers per arm ($N_{f}$) can be adjusted to maximize force intensity and travel range. These parameters are key to achieving the desired efficiency and functionality of the actuator within the defined constraints. Finally, design variables such as the electrode separation (d) are adjustable within design limits to meet specific performance requirements. This flexibility allows for fine-tuning of the design to achieve optimal travel range and force output. By categorizing and understanding these parameters, we can systematically optimize the design of the electrostatic comb drives, ensuring enhanced performance and reliability for silicon photonic applications.

#### 3.1.2. Mechanical Restoring Force and Traveling Range

To calculate the displacement of the comb drive designs, we assumed a uniform mechanical restoring force across all designs. This assumption ensures consistent and meaningful comparisons of force intensity, regardless of the specific restoring force used. For simplicity and to produce more representative and meaningful simulation results, we utilized the restoring force from the final fabricated design.

The mechanical restoring force in our study is based on the stiffness of a mechanical frame capable of holding two moving arms that actuate an asymmetric slab waveguide. This MEMS platform is designed for fabricating the waveguide on top. The slab waveguide and frame’s stiffness play a crucial role in determining the traveling range and the displacement of the comb drive actuators. Analyzing the frame’s properties allows accurate prediction of the force required for a given displacement. This analysis ensures that the optimized comb drive designs deliver the necessary performance metrics for actuating continuous stiff slab waveguides.

The simulated mechanical stiffness values and corresponding displacement calculations, detailed in Table 3 were derived from finite element analysis (FEM) simulations. These simulations provide a precise understanding of the frame’s behavior under various loading conditions. The dimensions of the frame and the resulting mechanical properties, crucial for our optimization process, directly influence the force intensity and traveling range of the comb drive actuators. Importantly, the frame dimensions were chosen based on fabrication feasibility, ensuring the practicality of the design. 

**Figure 6:**
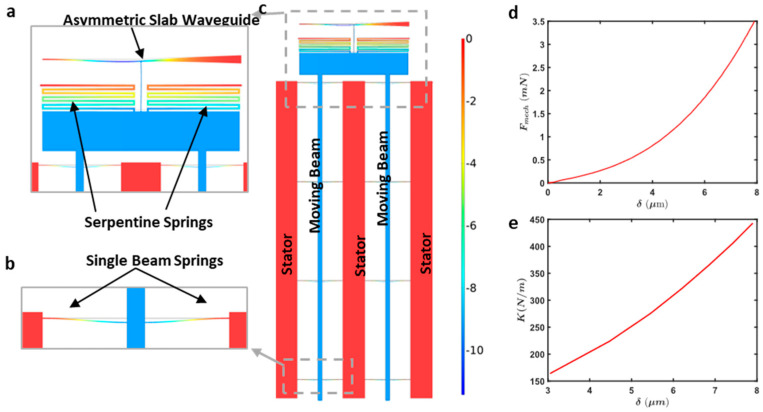
Mechanical stiffness and displacement analysis. (**a**): COMSOL simulation output showing the displacement profile of the frame under a 3.51 mN force load applied on mobile beams for a design with serpentine springs and an asymmetric slab waveguide. (**b**): Displacement profile for a design with single beam springs under the same loading conditions. (**c**): Enlarged view of the displacement in the moving beams and stators. (**d**): Plot of restoring force versus displacement, demonstrating the increasing displacement required for greater force. (**e**): Graph showing the mechanical stiffness versus displacement of the entire frame and slab waveguide platform, highlighting the non-linear stiffness characteristics.

By incorporating these mechanical properties into our analytical simulations, we ensure that the optimized comb drive designs are not only theoretically sound but also practically viable. Figure 6 illustrates the simulated displacement profiles and mechanical stiffness characteristics of the frame. The COMSOL simulation output (Figure 6a) shows the displacement profile of the frame under a 3.5 mN force load for a design with serpentine springs and an asymmetric slab waveguide. Figure 6b shows the displacement profile for a design with single beam springs under the same loading conditions. Figure 6c provides an enlarged view of the displacement in the moving beams and stators. The plot of restoring force versus displacement (Figure 6d) demonstrates the increasing displacement required for greater force. Finally, Figure 6e shows the mechanical stiffness versus displacement of the entire frame and slab waveguide platform, highlighting the non-linear stiffness characteristics.

In our device the total restoring force comes from three parts working together: the serpentine springs, the single-beam springs, and the asymmetric slab waveguide. The non-linearity we see in Figure 6c is mainly geometric. For small motions they mostly bend, so the response looks nearly linear. As the travel grows, the beams start to rotate and stretch (not just bend), and the slab behaves more like a plate that builds in-plane tension. That stress stiffening makes each extra micron of motion require more force. The changing geometry of the clamped-guided layout also shortens/lengthens effective lever arms as it moves, which adds to the stiffness increase. COMSOL large-deflection simulations using the measured, as-fabricated dimensions reproduce the same trend.

A note on slab symmetry: We use an asymmetric slab as a realistic photonic load [20], but the design method is general. The comb-array parameters (arm length/width, finger pitch, finger count) are chosen from the load’s stiffness and displacement curve. A symmetric slab would be treated the same after updating the stiffness. Any small in-plane moment from asymmetry was negligible in our tests; for very large arrays this will be analyzed separately.

#### 3.1.3. Mechanical Stability and Arm Width Optimization: Travel Range Sacrifice Ratio

Travel Range Sacrifice Ratio (TRSR) is a critical consideration in optimizing the mechanical stability of electrostatic comb drive actuators [16]. TRSR quantifies the trade-off between the actuator’s travel range and the permissible deflection of the arms to ensure operational stability and prevent side pull-in effects. Specifically, TRSR represents the percentage of the travel range that can be sacrificed to tolerate arm deflection along the arm, thereby preventing pull-in (the ideal TRSR is 0). The goal is to optimize the arm width ($d_{0}$) while maintaining mechanical integrity under maximum deflection ($D_{Max}\;$).

The maximum allowed deflection is calculated by considering the force applied by each comb finger along the arm. The overall deflection is derived from the summation of individual deflections caused by each finger’s force ($P_{i}$), distributed along the length of the arm ($L_{arm}$). The following equation shows the deflection of a cantilever at its tip with distributed point loads applied on it [51]:

(7) $$D_{Max}=\sum_{i}\frac{{P_{i}\;b}_{i}^{2}}{6\;E\;I}\;(3L_{arm}-b_{i})$$

In this equation, $P_{i}$ represents the force applied by the ith finger that is determined based on the total force needed to make 5 μm, movement of the comb frame presented in Figure 6. The $b_{i}$ denotes the distance from the arm anchor to the center of the ith finger, E is the modulus of elasticity of silicon, and I is the second moment of inertia for a rectangular cross-section based on the arm’s thickness (h) and width ($d_{0}$).

**Figure 7:**
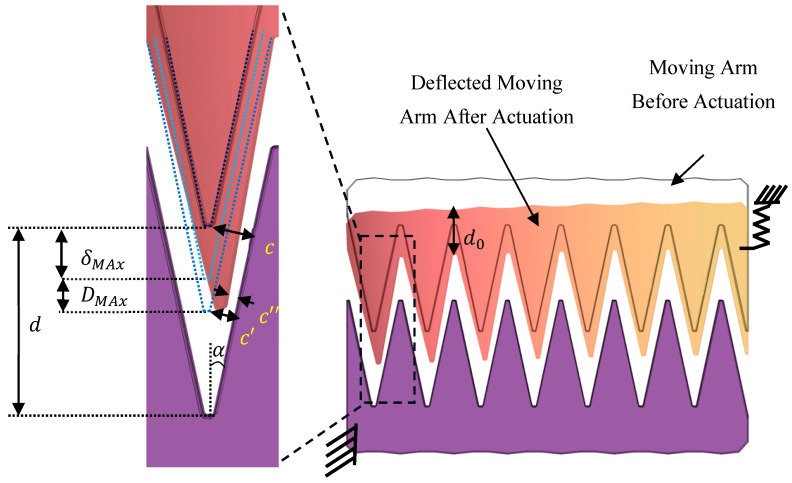
Schematic illustrating the reduction in electrode gap due to arm deflection in an electrostatic comb drive. The diagram shows the triangular finger configuration with parameters c, c′, and c″, indicating the initial gap, reduced gap due to travel range and deflection, and additional gap reduction from finger tilt due to deflection, respectively.

As illustrated in Figure 7 the critical parameter in determining the arm width is to ensure it is sufficiently wide to limit the deflection, thus preventing side pull-in effects. The arm width ($d_{0}$) must be optimized to keep the deflection within acceptable limits while maintaining the electrode gap above the pull-in threshold during maximum travel. To evaluate the impact of deflection on the electrode gap, the initial gap (c=dsinα) between electrodes is considered along with the reduction due to arm deflection ($D_{Max}$). The reduced gap (c″) at maximum travel range ($\delta_{Max}\;$) is calculated as:

(8) $$c_{\delta_{max\;}}^{\prime \prime }\;=\left(d\text{-}\;{\delta_{Max}-D}_{Max}\right)\sin\alpha \;-\frac{D_{max\;\;}}{2\sin\alpha }$$

In Equation (8) the term $\left(d\text{-}\;{\delta_{Max}-D}_{Max}\right)\sin\alpha$ reperesents the reduced gap between electrodes due to $\delta_{Max}$ traveling range (two-thirds of the initial gap) and the $D_{Max\;}$ deflection ($c^{\prime })$. Additionally, the $\frac{D_{max}}{2\sin\alpha }$ accounts for the further gap reduction due to rotation of the finger as the gap along the finger flank is not constant because of the tilt due to the arm deflection.

These calculations help determine the necessary arm width to ensure that the deflection does not lead to mechanical failure or side pull-in. The optimal arm width is selected to balance the force intensity, travel range, and mechanical stability.

By understanding and applying the travel range sacrifice ratio, we ensure the reliable operation of the comb drive actuators while optimizing the arm width for maximum performance. This approach enables the design of robust actuators capable of achieving high force intensity and extensive travel ranges without compromising mechanical stability.

**Figure 8:**
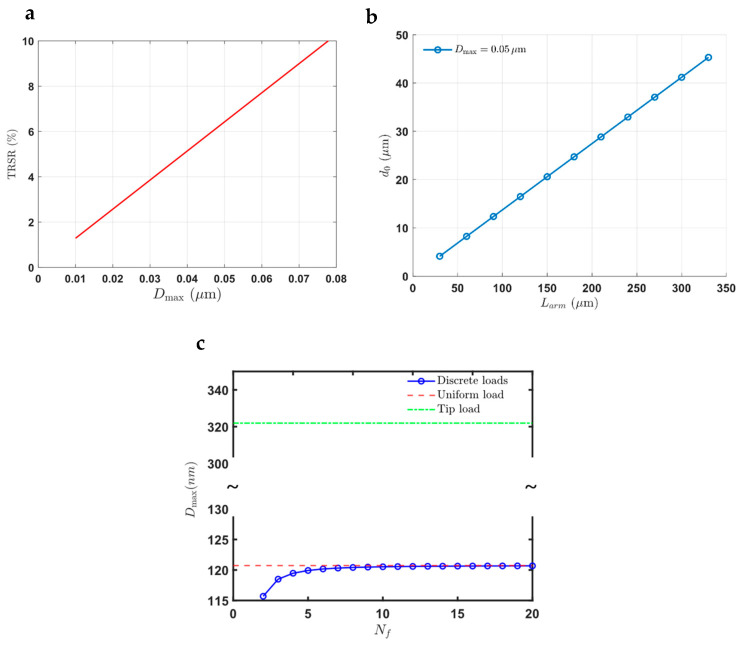
(**a**) Graph showing the travel range sacrifice ratio (TRSR) as a function of the maximum deflection ($D_{Max}$). The TRSR increases linearly with the maximum deflection, demonstrating the trade-off between the actuator’s travel range and its deflection stability. (**b**) Graph depicting the relationship between arm width ($d_{0}$) and arm length ($L_{arm}$) for a maximum deflection ($D_{Max}$) of 50 nm. The arm width increases with the arm length to maintain the specified maximum deflection, ensuring the mechanical stability of the actuator. (**c**) Tip deflection vs. total force per arm for three beam models on a single arm ($L_{arm}\;=\;187\;µm):$ single end load (upper bound), uniformly distributed load (lower bound), and discrete point loads at the finger locations ($N_{f}\;=\;12$ shown). Forces are swept from about 0–50 µN per arm, consistent with a total force up to ~5 mN for the full array.

In this work, we consider the maximum traveling range to be one-third of the initial gap, assuming linear stiffness and parallel plate behavior for simplicity. While the actual stiffness is slightly non-linear, this approximation provides a good representation for our purposes. Consequently, the reduced gap (c″) should be more than two-third of the initial gap to ensure effective operation. For this work, we have considered a 6% TRSR that leads to a $D_{Max}$ of 50 nm (Figure 8, based on Equation (8)). The graph for the associated $d_{0}$ to maintain the 50 nm $D_{Max}$ is shown in Figure 8b (based on Equation (7)).

Building on the TRSR-driven arm-width analysis above, we next benchmark the distributed per-finger load model against two standard cantilever cases at the same arm length ($L_{arm}\;=\;187\;µm$): (i) a single end-load, and (ii) a uniformly distributed load. Sweeping the total force per arm over the operating range (~0–50 µN) yields the tip-deflection curves in Figure 8c . As expected, the end-load gives the largest deflection (upper bound), the uniform load gives the smallest deflection (lower bound), and the discrete finger-load model lies in between. For $\;N_{f}\;\approx \;10–12,$ the discrete model closely tracks the uniform load curve across the operating force range. This supports using the discrete per-finger model in the main analysis, with the uniform load form serving as a simple check.

#### 3.1.4. Force Intensity in Different Arm Lengths (Analytical Calculations)

The analytical results are complemented by extensive simulations to verify the theoretical predictions and optimize the design parameters. These simulations account for the impact of different voltages and geometries on force intensity and travel range.

**Figure 9:**
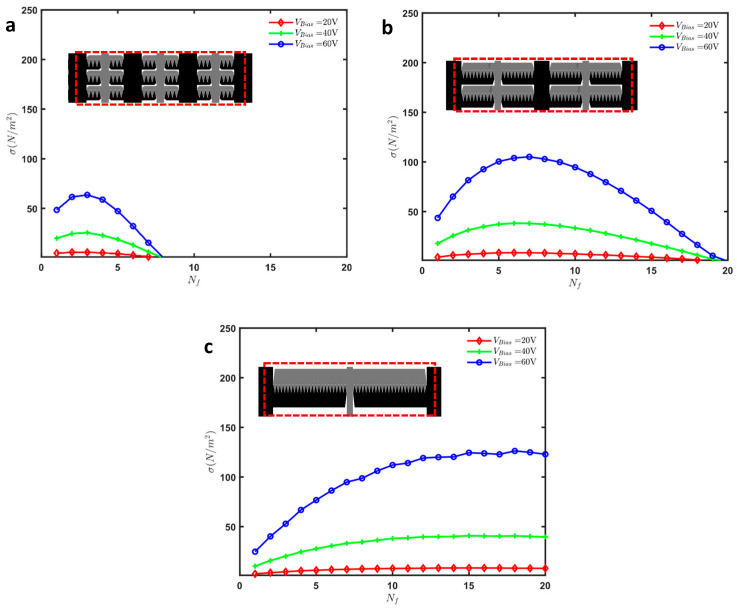
Force intensity of actuators with varying arm lengths and number of fingers per arm at different voltages. (**a**) Arm length of 50 µm, (**b**) Arm length of 125 µm, (**c**) Arm length of 350 µm. The results illustrate that increasing the arm length while reducing the number of stators enhances force intensity. Different voltage levels (20 V, 40 V, 60 V) are shown in red, green, and blue, respectively. The insects show the respective actuator designs.

Figure 9 provides a comprehensive comparison of force densities for different arm lengths and the number of fingers per arm at varying voltages. The configurations studied include arm lengths of 50 µm, 125 µm, and 350 µm, represented in Figure 9a–c. Figure 9a shows the force intensity for an arm length of 50 µm that is formatted in three sets of moving beams with 4 stators to hold the fixed beams. The force intensity peaks at approximately 60 N/m^2^ at 60 V, with a rapid decline as the number of fingers increases. This configuration demonstrates a high initial force intensity but is limited by the shorter arm length, leading to a quicker reduction in force intensity as more fingers are added.

Figure 9b presents the results for an arm length of 125 µm, formatted in two sets of moving beams and 3 stators with wider beams due to the longer arm length, reaching a higher peak of around 105 N/m^2^ at 60 V. The decline in force intensity is more gradual compared to the 50 µm arm length, suggesting better stability and efficiency over a wider range of finger numbers.

Figure 9c examines the force intensity for an arm length of 350 µm, with one set of moving beams and two stators and even wider beams, achieving the highest peak force intensity of approximately 125 N/m^2^ at 60 V. The extended arm length allows for a more stable and sustained force intensity across a larger number of fingers. The gradual decline indicates that this configuration can maintain high force intensity while accommodating more fingers, making it ideal for applications requiring high force over larger travel ranges.

The results illustrate that increasing the arm length enhances force intensity while reducing the number of stators. Longer arm lengths allow for more fingers to be effectively used without significant loss in force intensity. This optimization ensures that the actuator can achieve high force intensity and extensive travel range, crucial for continuous stiff slab waveguide actuation. By comparing these configurations, it is evident that a longer arm length coupled with an optimized number of fingers per arm provides the best performance in terms of force intensity and stability. However, the increase in force intensity is not linear with arm length; there is a plateau observed with longer arms.

Moreover, while longer arms need to be wider for mechanical stability, they generally show more force intensity. We will comprehensively study the arm length and force intensity relationship to determine the optimal length. It is important to note that very long freestanding MEMS structures may not be the best option due to practical limitations, and thus, the results will guide us in selecting the appropriate arm length for optimal performance.

**Figure 10:**
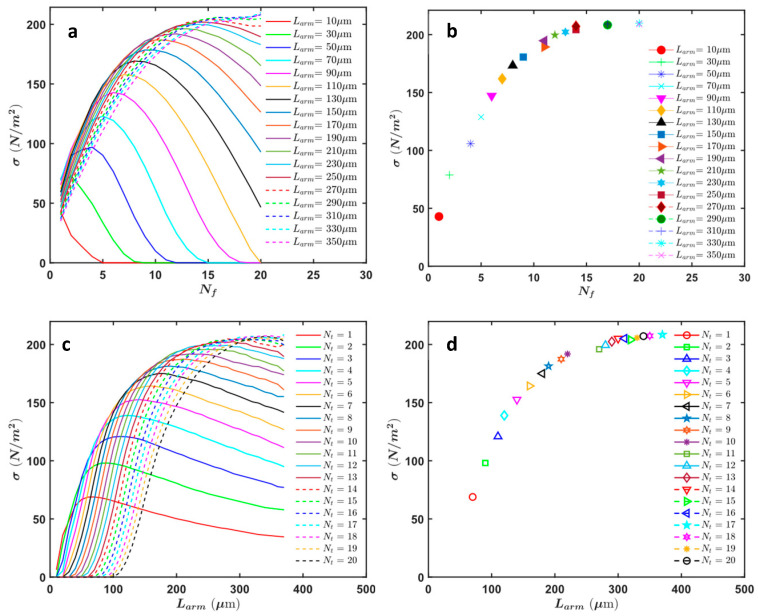
Comprehensive study of arm length and comb geometry focusing on optimized finger geometry for the highest force intensity while maintaining a specific traveling range. All results are for an applied voltage of 70 V. (**a**) Variation in force intensity with the number of fingers for different arm lengths, showing initial rise, peak, and decline in force intensity as the number of fingers increases. (**b**) Maximum force intensity values from (**a**) for each arm length, highlighting the optimal number of fingers for the highest force intensity for each arm length. (**c**) Force intensity as a function of arm length for different numbers of fingers, with each curve representing a specific number of fingers. (**d**) Maximum force densities from (**c**) for each finger configuration, indicating the optimal arm length for each number of fingers, showing the need to balance arm length and finger count for optimal performance.

Figure 10 provides a comprehensive study of the arm length and comb geometry, focusing on the optimized finger geometry for the highest force intensity while maintaining a specific traveling range. All the results shown in Figure 10 are for an applied voltage of 70 V. Figure 10a shows the variation in force intensity with the number of fingers for different arm lengths. As the number of fingers increases, the force intensity initially rises, peaks, and then declines. Each curve represents a different arm length, indicating how the force intensity changes with increasing numbers of fingers. Shorter arm lengths achieve peak force intensity at lower numbers of fingers, whereas longer arm lengths can support more fingers before the force intensity starts to decline.

Figure 10b marks the maximum force intensity values from Figure 10a for each arm length. The optimal number of fingers that produce the highest force intensity for each arm length is highlighted. It is observed that longer arm lengths achieve higher maximum force intensities, demonstrating the benefit of extended arms for applications requiring greater force.

Figure 10c illustrates the force intensity as a function of arm length for different numbers of fingers. Each curve represents a specific number of fingers, showing how force intensity varies with different arm lengths. The force intensity increases with arm length for all configurations, with varying rates of increase depending on the number of fingers.

Figure 10d marks the maximum force densities from Figure 10c for each finger configuration, indicating the optimal arm length for each number of fingers. The optimal arm length increases with the number of fingers, highlighting the need to balance arm length and finger count for optimal performance.

**Figure 11:**
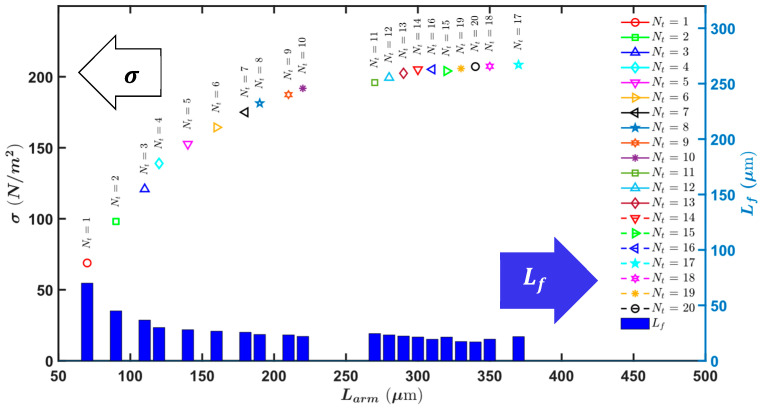
This figure illustrates the force intensity (σ) as a function of arm length ($L_{arm}$) for various numbers of fingers ($N_{f}$). The different colored markers represent different numbers of fingers, and the blue bars indicate the corresponding finger pitch ($L_{f}$). The figure demonstrates that the finger pitch tends to have a consistent value across optimal configurations. All the results shown are for an applied voltage of 70 V. After $N_{f}=11$, the force intensity does not exhibit significant changes, suggesting that beyond this point, the force intensity stabilizes regardless of the number of fingers and the specific finger pitch. Each combination of finger number and finger pitch results in a particular optimal arm length. This analysis emphasizes the critical role of finger pitch in achieving optimal force intensity and mechanical stability in electrostatic comb actuators.

In addition to the analysis presented in Figure 10, Figure 11 provides further insights by incorporating the finger pitch for each data point (all the results shown in Figure 11 are for an applied voltage of 70 V). This additional parameter is crucial as it reveals that the finger pitch tends to have a consistent value across optimal configurations.

The figure shows that after $N_{f}=11$, the force intensity does not exhibit significant changes, suggesting that beyond this point, the force intensity stabilizes regardless of the number of fingers and the specific finger pitch. The data points indicate that each combination of finger number and finger pitch results in a particular optimal arm length.

This additional analysis emphasizes the importance of finger pitch in achieving optimal force intensity and mechanical stability. The consistency of the finger pitch across different configurations highlights its critical role in the design of electrostatic comb actuators. The figure also demonstrates that for configurations beyond $N_{f}=11$, the actuator design must consider both the number of fingers and the finger pitch to maintain high performance and stability.

These findings provide valuable insights into the design parameters that affect force intensity and actuator performance. The results guide the development of optimized electrostatic comb actuators with a specific focus on balancing arm length, number of fingers, and finger pitch to achieve the desired force intensity and mechanical stability. These optimizations are crucial for applications in continuous, stiff slab waveguide actuation, enhancing the performance and reliability of silicon photonic devices.

### 3.2. Arm Geometry Optimization

In this section, we delve into the optimization of arm geometry to achieve significant footprint savings without compromising mechanical stiffness and performance. A key aspect of our design is the reduction in separation between cells by tapering the arms. This approach ensures that the force generated by the electrostatic actuators is not adversely affected by the interactions between cells while simultaneously minimizing the overall footprint of the device.

**Figure 12:**
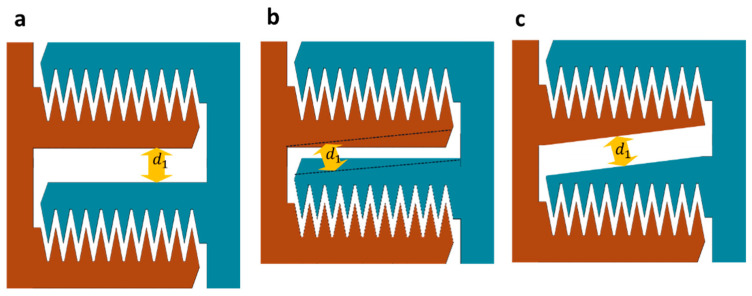
Arm tapering to reduce cell-to-cell separation while preserving inter-cell spacing. (**a**) Baseline layout with straight arms; the inter-cell spacing is $d_{1}\;$(nominally 17 µm). (**b**) Tapered-arm concept: the arms narrow along their length to bring the arrays closer while maintaining the local spacing $d_{1}$ between adjacent cells. (**c**) Realized tapered layout that reduces footprint; $d_{1}$ is preserved to prevent unwanted electrostatic attraction and to maintain mechanical stability and efficient force transmission.

**Figure 13:**
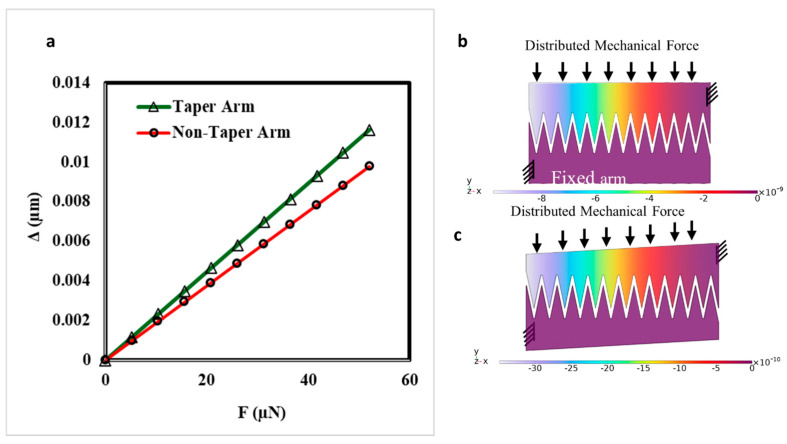
Comparison of taper deflection to non-taper deflection. (**a**) Tip displacement (Δ) versus applied force (F) for tapered (27 reduced to 21 μm) and non-tapered (27 μm) arms; the tapered arm shows slightly larger Δ) over 0–50 μN per arm (≈0–5 mN for 96 arm pairs). (**b**) Displacement field of the non-tapered arm under the simulated loading; the color scale (see bar) encodes the displacement magnitude and the arrows mark where the distributed mechanical loads are applied. (**c**) Displacement field of the tapered arm under the same loading; colors and arrows have the same meaning as in (**b**). In the tapered arm, the width is reduced from 27 μm to 21 μm over the arm length. Numerical results show that the displacement (Δ) at the tip of the tapered arm due to bending is slightly higher than that of the non-tapered arm for the force range applied to one arm. A distributed force equivalent to the force applied to each arm was applied on top of the arm to observe the deflection behavior.

As depicted in Figure 12, the arm is tapered while maintaining a 17 µm inter-cell spacing, keeping inter-cell electrostatic attraction. This careful design consideration prevents any negative impact on the electrostatic force due to unwanted interactions between adjacent cells. By tapering the arms, we ensure that the mechanical stiffness of the actuators remains intact, allowing for efficient force transmission and reliable actuation. The reduction in cell separation achieved through this tapering technique contributes significantly to the compactness of the overall design. This optimization is particularly beneficial for applications where space constraints are critical, such as in densely packed photonic circuits and other integrated photonic devices. Furthermore, the optimization of arm geometry not only enhances the force intensity and travel range but also improves the mechanical stability of the actuators. By ensuring that the arm length and tapering are precisely controlled, we maintain the robustness of the actuators, preventing issues such as lateral instability and pull-in effects. In addition, we have numerically calculated the taper deflection compared to the non-tapered ones. We tapered the 27 µm arm width down to a 21 µm arm width with the dimensions provided in Table 2. The numerical results show that the bending at the tip of the tapered arm (Δ) is slightly higher but negligible, indicating that they have essentially the same performance as the non-tapered arm for the range of force applied to one arm (Figure 13).

Here, we considered that a 96-pair of arms will generate a force between 0 and 5 mN, with the deflection at the tip of the arms depicted in Figure 13. Moreover, the numerical results show less deflection compared to the analytical results of a simple cantilever deflection that will discuss in upcoming sections. This is due to the fingers introducing a larger second moment of area, providing better performance and a safer margin. Overall, the optimized arm geometry, combined with the tapering strategy, provides a highly efficient and compact solution for electrostatic comb drive actuators, making them suitable for a wide range of applications in silicon photonics and beyond.

### 3.3. Analytical Calculations and Numerical Simulation of Selected Design

#### 3.3.1. Analytical Calculations

**Figure 14:**
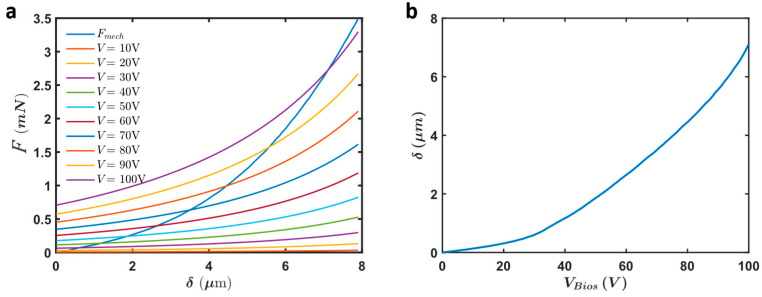
(**a**) Analytical results showing the electrostatic force ($F_{elec}$) as a function of displacement (δ) for various applied voltages (V), alongside the mechanical restoring force ($F_{mech}$). The intersection points where $F_{elec}$ equals $F_{mech}$ indicate the stable operating points of the actuator, determining its displacement. (**b**) Analytical results depicting the displacement (δ) as a function of the applied bias voltage ($V_{bias}$). The displacement increases non-linearly with the applied voltage, indicating the actuator’s performance under different electrical conditions.

The analytical calculations provided for the selected device in the previous section aimed to determine the electrostatic force and displacement of the actuator under various voltages. These calculations were essential in understanding the force output, displacement, and stability of the actuators across different operational conditions. The results from these calculations revealed key insights into the actuator’s performance. The electrostatic force ($F_{elec}$) and mechanical restoring force ($F_{mech}$) were calculated for different applied voltages (Figure 14a). The stable operating points of the actuator are determined where the mechanical restoring force equals the electrostatic force. This balance point dictates the displacement of the actuator (Figure 14b). By analyzing these stable points, we can predict the performance of the actuator under various applied voltages.

#### 3.3.2. Numerical Simulations

To validate our analytical calculations, we conducted a comprehensive Finite Element Method (FEM) simulation using COMSOL Multiphysics 6.1. This simulation was meticulously designed to reflect the real-world behavior of the optimized triangular finger comb drives.

The simulation setup was carefully crafted to replicate the actual comb drive structure, particularly the chamfered arm and finger design. This attention to detail ensured that the physical interactions within the device were realistically modeled. Silicon was selected for the electrodes, leveraging its common application in MEMS technology, while air served as the dielectric medium. The mechanical and electrical properties of these materials were derived from the COMSOL library.

The model integrated multiple physics interfaces, including solid mechanics, electrostatics, moving mesh, and electromechanical force physics. This comprehensive approach allowed for a detailed analysis of the complex interactions between mechanical and electrical forces within the device. The simulation utilized a mesh composed of tetrahedral elements, with settings optimized to balance accuracy and computational efficiency. The mesh configuration included a maximum element size of 7.75 µm, a minimum element size of 0.56 µm, and a maximum growth rate of 1.4.

**Figure 15:**
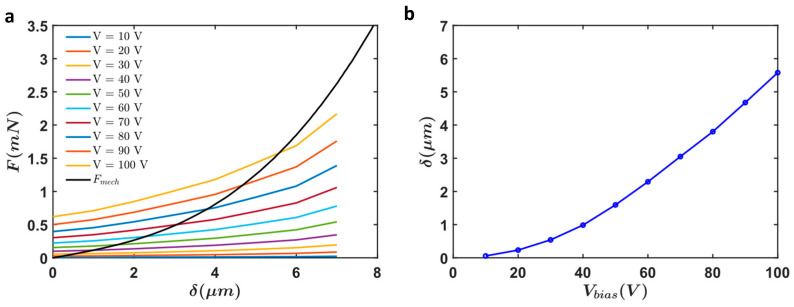
(**a**) Numerical simulation results showing the force (F) as a function of displacement (δ) for various applied voltages (V). The mechanical restoring force ($F_{mech}$) is also plotted for comparison. (**b**) Analytical results depicting the displacement (δ) as a function of the applied bias voltage ($V_{bias}$). The displacement increases non-linearly with the applied voltage, indicating the actuator’s performance under different electrical conditions.

Focusing on the specific configuration of 12 fingers per arm, aligned with the selected device parameters detailed in the previous section, the setup enabled a thorough evaluation of the comb drive actuator’s performance under operational conditions. This simulation provided critical validation for our analytical predictions, confirming the robustness and effectiveness of the proposed design (Figure 15).

### 3.4. Design Selection for Fabrication

To choose a design for fabrication, Figure 11 provided the highest force intensity for each configuration. However, from Figure 10c, it is evident that many configurations have approximately the same force intensity, so it was not necessary to choose strictly from the options proposed in Figure 11. In Table 4, all configurations with a force intensity above 180 N/m^2^ for the 70 V bias voltage are summarized (extracted from Figure 10c). The data is categorized based on the arm length, and for each arm length, the best configurations of ($N_{f},L_{f})$ and the corresponding force intensity range are provided. For enhanced mechanical stability, the design with the minimum arm length that still has high force intensity was selected. Specifically, we chose the configuration from the first row with an arm length of 180 µm, 12 fingers, and a finger length of 15 µm, which has a force intensity of $184.8\;N/m^{2}.$ All the details of fabricated device dimensions are in Table 2. For the fabrication, we calculated that a total of 48 cells (96 pairs of arms) will be needed to achieve a 5 µm traveling range for the given slab waveguide presented in Table 2.

### 3.5. Testing and Validation

#### 3.5.1. SEM Characterization of Fabricated Electrostatic Comb Drive Actuators

**Figure 16:**
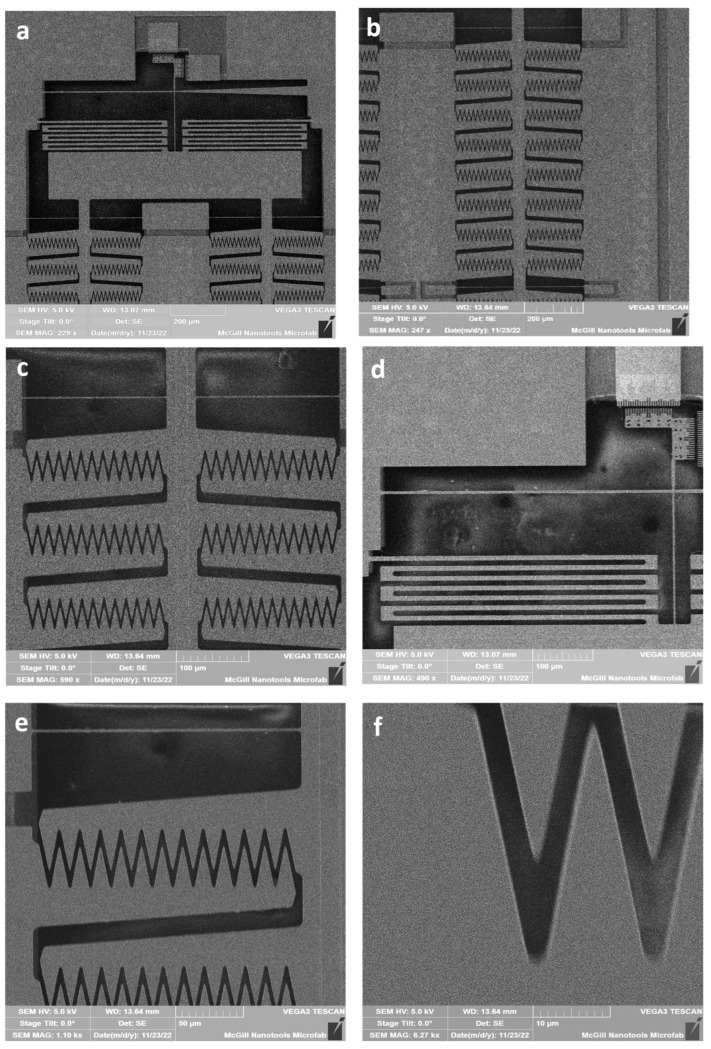
SEM images of the fabricated electrostatic comb drive actuators. (**a**) Top view of the device showing the asymmetric slab waveguide, vernier scale for movement measurement, serpentine spring for electrical connections, single beam springs, and actuator arms. (**b**) Detailed view of a section of the actuator arrays, illustrating the repetitive structure of the actuator cells. (**c**) Close-up of one part of the actuator repeated cell, emphasizing the triangular finger configuration and alignment. (**d**) Zoomed view of the serpentine spring and connection beam between the slab waveguide and the actuator, highlighting the mechanical and electrical integration. (**e**) Detailed view of one actuator arm and the separation between cells, showing the structural design and spacing. (**f**) High-magnification view of a single triangular finger, displaying the precision in fabrication and the small gap necessary for effective electrostatic actuation.

The fabricated electrostatic comb drive actuators were characterized using Scanning Electron Microscopy (SEM) to validate the precision and integrity of the design. The SEM characterization was performed using a VEGA3 TESCAN (TESCAN, Brno, Czech Republic). Figure 16a shows the top view of the device, featuring the asymmetric slab waveguide, a vernier scale for measuring movements, serpentine springs for electrical connections, single beam springs, and actuator arms. This comprehensive view highlights the detailed design necessary for integrating mechanical and photonic components effectively.

Detailed SEM images of the actuator arrays and their components provide further insights. Figure 16b presents a close-up of a section of the actuator arrays, illustrating the repetitive structure essential for achieving high force intensity and uniform actuation. Figure 16c zooms in on a part of the actuator repeated cell, emphasizing the alignment and precision of the triangular fingers. Figure 16d focuses on the serpentine spring and the connection beam between the slab waveguide and the actuator, highlighting the mechanical and electrical integration necessary for device functionality. Figure 16e provides a detailed view of one actuator arm and the separation between cells, showcasing the structural design and spacing required for stable operation. Finally, Figure 16f offers a high-magnification view of a single triangular finger, demonstrating the fabrication precision and the small gap critical for effective electrostatic actuation.

#### 3.5.2. Testing Results

**Figure 17:**
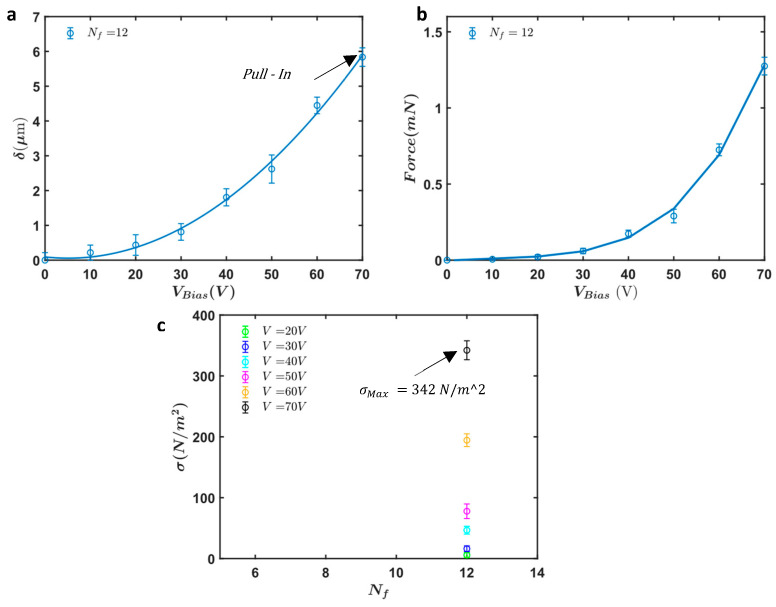
Experimental validation of the optimized electrostatic comb drive actuators. (**a**) Measured displacement (δ) of the actuator with $N_{f}=12$ fingers as a function of the applied bias voltage ($V_{Bias}$). The displacement increases non-linearly, with a significant rise near 70 V where a pull-in event occurs, achieving a maximum displacement of approximately 6 μm. Error bars indicate measurement consistency. (**b**) The force output of the actuator shows a non-linear increase with applied voltage, reaching approximately 1.3 mN at 70 V. Calculations were based on the effective stiffness of the mechanical frame derived from SEM images and COMSOL simulations. (**c**) Force intensity (σ) of the fabricated design with 12 fingers for applied voltages from 20 V to 70 V. The maximum force intensity achieved is 342 N/m^2^ at 70 V. Error bars indicate the standard deviation based on three repeated measurements.

Figure 17a shows the measured displacement (δ) of the actuator with $N_{f}=12$ fingers as a function of the applied bias voltage ($V_{Bias}$). The displacement increases non-linearly with the applied voltage, exhibiting a significant rise as the voltage approaches 70 V. At this voltage, the actuator experiences a pull-in event, achieving a maximum displacement of approximately 6 μm. The error bars indicate the consistency and reliability of the measurements across multiple tests.

**Figure 18:**
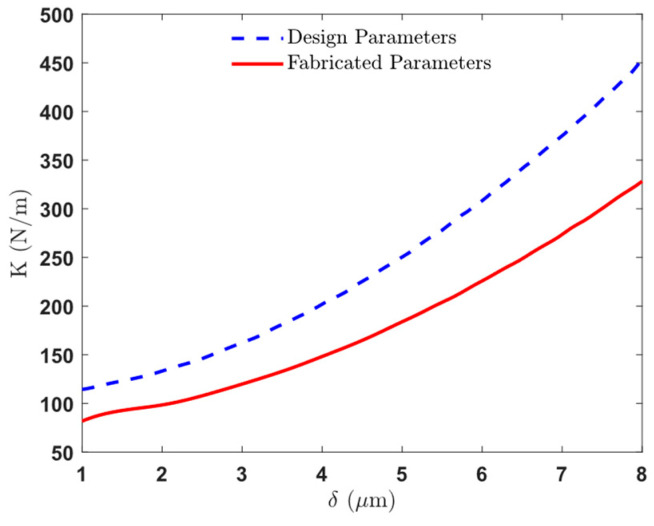
Comparison of stiffness (K) as a function of displacement (δ) for design parameters (blue) and fabricated parameters (red). The discrepancy highlights the impact of over-etching on mechanical stiffness.

The force output generated by the actuator is depicted in Figure 17b. Similarly to the displacement results, the force output shows a non-linear increase with the applied voltage. The maximum force recorded is approximately 1.3 mN at 70 V. To obtain these force measurements, we calculated the force based on the stiffness of the mechanical frame, which includes all single beam springs, serpentine springs, and the slab waveguide. We utilized the fabricated dimensions obtained from SEM images and conducted numerical simulations using COMSOL to calculate the effective stiffness of the entire frame (Figure 18). Using this effective stiffness, we accurately determined the force output, confirming the effectiveness of the electrostatic actuation mechanism.

Figure 17c illustrates the force intensity (σ) of the fabricated design that has 12 fingers for various applied voltages ranging from 20 V to 70 V. The data reveals that the force intensity increases with the applied voltages. The highest force intensity achieved is $342\;N/m^{2}$ at an applied voltage of 70 V.

### 3.6. Comparison of Simulation and Fabrication Results

After running the experiments, we observed some discrepancies between the experimental results and the simulations we conducted earlier. We then investigated these discrepancies to determine their root cause.

Spring Over-Etching: One discrepancy resulted from over-etching during the fabrication process, altering the dimensions of the springs and affecting the overall mechanical stiffness of the system. To address this, SEM images were analyzed to determine the actual fabricated dimensions, which were then incorporated into the finite element model (FEM) to calculate the corrected stiffness. This corrected stiffness was subsequently used to calculate the force and force intensity accurately.

The comparison of stiffness between design parameters and fabricated parameters is illustrated in Figure 18, showing the significant impact of over-etching on mechanical stiffness.

Electrode Dimension Changes: Another difference observed from the design dimensions was the electrode over-etching. To account for this, SEM images were utilized to measure the fabricated dimensions, which showed both widened finger angles (due to over-etching) and discrepancies in electrode gap sizes. SEM measurements revealed that the finger angle (alpha) is 11.76 degrees and the gap (d) is 14.5 µm. After applying these corrections, the simulation results aligned closely with the experimental data, validating the effectiveness of the optimization process and the accuracy of the corrected model.

**Figure 19:**
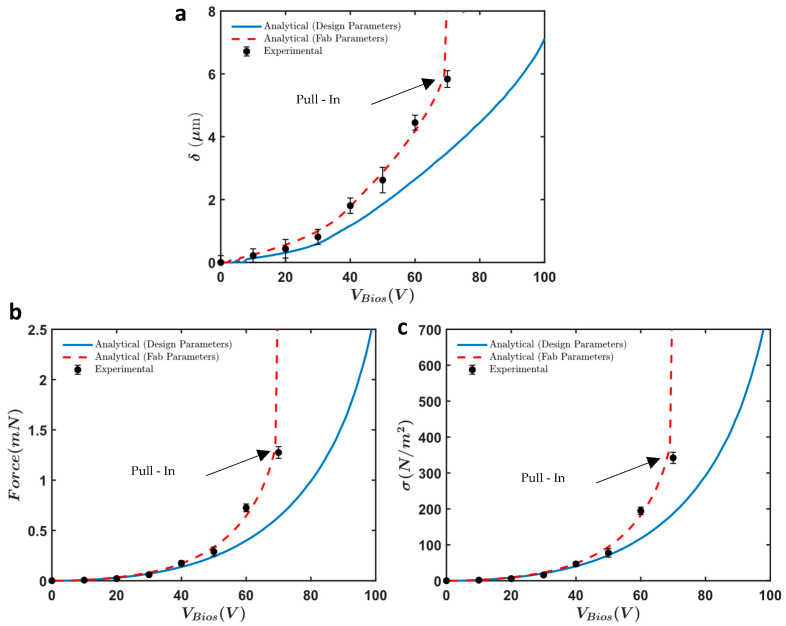
Comparison of simulation and experimental results. (**a**) Displacement (*δ*) vs. applied bias voltage ($V_{Bias}$) for analytical design parameters (blue solid line), analytical fab parameters (orange dashed line), and experimental results (black points). The corrected model aligns closely with the experimental data, showing a significant pull-in event near 70 V. (**b**) Force vs. applied bias voltage, the corrected model closely matches the experimental data. (**c**) Force intensity (σ) vs. ($V_{Bias}$), demonstrating the accuracy of the corrected model compared to experimental measurements. The highest force intensity achieved experimentally is 342 N/m^2^ at 70 V, consistent with the corrected analytical model. Error bars indicate the standard deviation based on three repeated measurements.

Figure 19 illustrates the comparison between the analytical (design and fabrication parameters) and experimental results for displacement, force output, and force intensity. The experiments showed more displacement and force compared to the initial simulations due to two main reasons. First, the springs and slab waveguide were softer than anticipated because of over-etching during fabrication. Second, the gap between the electrodes was slightly smaller than the design parameters, resulting in increased force. Both the softening of the springs and the narrowing of the gap contributed to the observed increase in displacement and force.

### 3.7. Fabrication Tolerances

**Figure 20:**
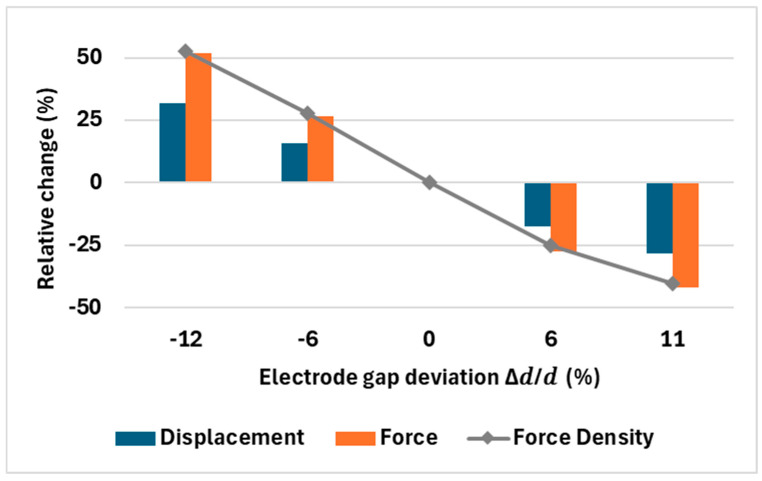
Sensitivity of actuator metrics to electrode-gap deviation. Relative change (%) in displacement, force, and force intensity versus electrode-gap deviation (Δd/d = −12%, −6%, 0%, +6%, +11%). Negative *x*-axis values mean the fabricated gap is smaller than the design; positive values mean it is larger. Consistent with the inverse-square dependence on gap, force intensity is the most sensitive (for example, a −6% gap gives roughly +25–30%, while a +6% gap gives roughly −25%), whereas displacement varies more moderately due to the frame stiffness.

In practice several dimensions can drift during fabrication and all of them affect performance. Among these, the electrode gap d and the overall frame/spring stiffness have the largest impact. Gap directly sets the electrostatic force (strong $1/d^{2}$ dependence), while frame stiffness sets how much of that force converts to travel. To quantify the dominant effect, we varied d by ±12% around nominal (Table 2) and plotted the relative changes in displacement, force, and force density (Figure 20). As expected, force intensity is most sensitive to d. Displacement moves more moderately. The stiffness dependence itself was analyzed in (Figure 18), showing how the restoring force varies with fabrication discrepancy. Simultaneous variation in all parameters is difficult to simulate compactly, but bracketing the design with d sweeps and the measured stiffness provides a good tolerance estimate. In practice, we use the first fabrication run to calibrate the model (SEM-measured as-built gaps and widths) and then pre-compensate the mask; subsequent runs can hit a specific target with high accuracy in future fabrication iterations.

## 4. Discussion

This paper presents a novel approach for actuating a monolithic component that requires higher force using an array of optimized electrostatic comb actuators. For applications requiring higher forces, arrays of repetitive comb arms are necessary. This study includes the optimization of these arrays, determining the best configuration to maximize force intensity and travel range while ensuring mechanical stability and preventing pull-in effects. The study does not merely aim to increase the force output but focuses on enhancing force intensity, ensuring that the actuators generate significant force without requiring a very large footprint while maintaining the travelling range. This is critical as it allows for efficient and compact actuator designs, avoiding the need for excessively large actuators to achieve higher forces.

A key aspect of this optimization is maintaining a substantial traveling range. Specifically, the optimization process targeted a displacement of 5 µm, ensuring that the actuators can achieve the necessary movement without sacrificing performance. By avoiding the reduction in the gap to increase force and decrease the actuator footprint, the design maintains both high force intensity and adequate travel range. The proposed design is specifically tailored to provide robust and reliable actuation for monolithic components in silicon photonics, paving the way for advanced tunable photonic devices.

### 4.1. Comparison with Previous Studies

Previous research on electrostatic comb drives has predominantly focused on optimizing specific aspects of the comb drive design without a comprehensive consideration of both footprint and mechanical stability. These studies often worked within fixed arm lengths, which imposed limitations on the achievable force output and travel range. Our study introduces a new design method by removing these arm length restrictions and fully optimizing both the arm size and finger geometry, particularly using triangular fingers.

This comprehensive optimization approach, which includes finger geometry, arm length, the number of fingers per arm, and finger pitch, results in a significant enhancement in force intensity and travel range. Specifically, by focusing on the optimal configuration of comb structures with triangular fingers, our design is able to generate the required force while maintaining a specified displacement range.

### 4.2. Detailed Analysis of Results

The analytical calculations highlighted the critical interplay between electrostatic force and mechanical restoring force in determining the actuator displacement under various voltages. The balance point, where the electrostatic force equals the mechanical restoring force, dictates the stable operating points of the actuator. These stable points were instrumental in predicting actuator performance, and this was further corroborated by numerical simulations. The simulations demonstrated high precision in modeling the physical interactions of the comb drives, accurately reflecting their real-world behavior.

The experimental results closely aligned with the theoretical predictions, validating our optimization process. However, discrepancies arose due to fabrication variations, such as over-etching and changes in electrode dimensions. To address these issues, we conducted detailed SEM analyses and applied correction functions to adjust for these fabrication discrepancies. This ensured that our simulation results accurately matched the experimental data, confirming the robustness and effectiveness of the proposed design.

Moreover, the results revealed a plateau effect in force intensity with increasing arm length. This phenomenon suggests that beyond a certain point, extending the arm length does not significantly enhance force intensity (Figure 11). Instead, it may introduce mechanical instability due to the potential for out-of-plane deflection caused by the weight of longer arms. As a result, it is preferable to use shorter arm lengths to maintain mechanical stability while still achieving high force intensity.

Figure 19 provides comprehensive comparisons, illustrating the consistency between analytical, numerical, and experimental data. These figures underscore the effectiveness of our optimization strategy, ensuring that the comb drive actuators perform reliably under the specified operational conditions. The key performance metrics for the optimized electrostatic comb drive actuators include force intensity, travel range, and mechanical stability. Our design achieved a force intensity of 342 N/m^2^ at 70 V, with a travel range of 6 µm. These metrics highlight the significant improvement in actuator performance compared to previous designs.

### 4.3. Fabrication Challenges and Solutions

During the fabrication process, several challenges were encountered, such as over-etching and variations in electrode dimensions. These issues affected the mechanical stiffness and electrostatic force calculations. By conducting detailed SEM analyses and applying correction functions, we were able to adjust our simulations to accurately reflect the fabricated dimensions. This ensured that our theoretical models aligned closely with the experimental results, validating the robustness of our design.

### 4.4. Implications for Silicon Photonics

The optimized electrostatic comb drive actuators have significant implications for silicon photonics. Their high force intensity and compact footprint make them ideal for applications requiring precise and reliable actuation of monolithic components. This study paves the way for advanced tunable photonic devices, potentially enhancing the performance and functionality of silicon photonic systems. Specifically, these actuators are particularly well-suited for optical beam steering in in-plane silicon photonics applications. The ability to achieve substantial force output without sacrificing travel range or mechanical stability is critical for the development of next-generation optical devices. By enabling precise control over the direction of light within the silicon photonic chip, these actuators can significantly improve the performance and adaptability of integrated optical systems.

### 4.5. Limitations and Future Work

While this study achieved significant advancements, there are limitations to consider. One major limitation is the fabrication discrepancies encountered, such as over-etching, which affected the precise dimensions of the actuators. To address this, future work can utilize our model to intentionally adjust design parameters to achieve the exact desired dimensions post-fabrication [16]. Additionally, further efforts can be directed towards refining the fabrication process itself to minimize over-etching effects and enhance overall accuracy.

Moreover, there is potential for further improvement in the actuator designs, particularly in the spring configurations. By optimizing the spring configurations, we can enhance both force intensity and mechanical stability, providing even more robust performance. Future research will also explore alternative materials and fabrication techniques to further enhance the performance of these actuators.

Integrating these optimized actuators into complex photonic systems remains a key area of research, with the goal of significantly improving the functionality and efficiency of silicon photonic devices. This will pave the way for even more advanced tunable photonic devices, enhancing the capabilities of silicon photonics technology.

### 4.6. Conclusion of Discussion

In summary, this study has demonstrated significant advancements in the optimization of electrostatic comb drive actuators for monolithic components requiring higher force intensity and travel range. By addressing fabrication discrepancies and applying correction functions, we have ensured that our theoretical models align closely with experimental results, confirming the robustness and effectiveness of the proposed design. The key performance metrics achieved highlight the success of our comprehensive optimization approach, resulting in actuators that provide high force without requiring a large footprint. This optimization is crucial for creating compact and efficient designs, essential for actuating stiff monolithic components in silicon photonics. Future work will continue to refine the fabrication process and actuator designs, further improving performance and enabling advanced tunable photonic devices.

## 5. Conclusions

This paper presents a novel optimization strategy for electrostatic comb drive actuators designed to meet the high force intensity and compact footprint requirements of stiff monolithic slab waveguides in silicon photonics. Moving beyond optimization of one comb in isolation, we co-optimized a repeatable, footprint-optimized array unit cell (arm length and width, finger pitch, and finger count) and validated it against an asymmetric slab waveguide load. This raised the maximum pre-pull-in force density from about 200 N m^−2^ at 100 V in our earlier design to about 342 N m^−2^ at 70 V here, while extending the travel from about 3.5 µm to about 6 µm. The higher performance at lower voltage comes from the array layout and the arm co-design, with analytical and numerical models closely matching the measurements. After fabrication, we measured the actual device dimensions with SEM and updated the model; the predictions then matched the measurements well

The optimized actuators are particularly effective for applications in optical beam steering within in-plane silicon photonics, providing precise and reliable actuation for stiff monolithic components. This study lays the foundation for future research focused on refining fabrication processes, enhancing spring configurations, and integrating these actuators into complex photonic systems. Our work paves the way for the development of advanced tunable photonic devices, significantly enhancing the performance and functionality of silicon photonics technology.

## Figures and Tables

**Table 2:** Design parameters and fabricated device dimensions. This table summarizes the key parameters used in the design and fabrication of the electrostatic comb actuator devices. It includes descriptions, types, limitations, and values for each parameter. The parameters are categorized into fabrication process constraints (FPC), design variables (DV), design restrictions (DR), and optimization parameters (OPT).

Symbol	Description	Type	Limitations	Fabrication Value
h	Element thickness	FPC	SOI silicon layer thickness	10 µm
c	Finger separation (Dependent)	FPC	c=dsin(α)≥3 μm	3.51 µm
$L_{c}$	Connection beam length between arms and mobile or fixed beams	FPC	≥2 μm	5 µm
$L_{s}$	Stator length	FPC	Deep trench under etch	200 µm
α	Triangular finger half angle	FPC	Losing sharp tips	10°
d	Electrode separation along the Y direction	DV	Proportional to the traveling range	17 µm
$d_{0}$	Arm Width	DR	Bending and side pull-in	27–21 µm *
$d_{1}$	Distance to the next pair of electrodes	DR	Minimize the Electrostatic Force	17 µm
$L_{m}$	Moving rail length	DR	Electrode lateral instability	40 µm
$D_{max}$	Maximum bending deflection	DR	Electrode lateral instability	0.05 µm
$L_{f}$	Finger pitch	OPT	-	15 μm
$N_{f}$	Number of fingers in each arm	OPT	-	12
$L_{arm}$	Arm Length	DP	$L_{arm}=N_{f}L_{f}$	187.5 μm
$d_{L}$	projected TRI electrode length on *Y*-axis	DP	$d_{L}=\frac{L_{f}}{2\tan\alpha }$	
$L_{cell}$	Cell length	DP	$L_{cell}=L_{arm}+{2L}_{c}+\frac{1}{2}L_{s}+\frac{1}{2}L_{m}$	
$W_{cell}$	Cell width	DP	$w_{cell}=2d_{0}+d+d_{L}+d_{1}$	
N_cell	The number of cells in the comb drive	DR	Device maximum allowed footprint	48

* Tapered arm narrowing from 27 µm to 21 µm.

**Table 3:** Key Parameters and Values for Slab Waveguide and Comb Frame Structure.

	Description	Value
	**Slab Waveguide**	
$w_{1}$	Rectangular beam width of the slab (left side)	5 µm
$w_{2}$	Trapezoid beam width of the slab (right side)	40 µm
$L_{1}$	Rectangular beam length	500 µm
$L_{2}$	Trapezoid beam length	500 µm
h	Beam thickness	10 µm
δ	Deflection of the beam at middle	5 µm
F	Force applied at middle	3.5 mN
E	Young’s modulus (silicon <110>)	169 GPa
	**Comb Frame Structure**	
$W_{s1}$	Width of single beam springs	2 µm
$W_{s2}$	Width of serpentine springs	9 µm
$l_{s2}$	length of serpentine springs	467 µm
$l_{s1}$	Length of single-beam springs	197 µm
$P_{s2}$	The pitch of the serpentine springs	19 µm
$L_{s}$	Stator beam length	200 µm
$L_{m}$	Mobile beam length	40 µm
h	Element thickness	10 µm

**Table 4:** Summary of Configurations with Force intensity Above 180 N/m^2^ at 70 V Bias Voltage. This table categorizes various configurations based on Figure 10c in terms of arm length ($L_{arm}$) and lists the best configurations for each arm length, showing the number of triangular fingers ($N_{f}$) and finger length ($L_{f}$). The corresponding force intensity ranges (σ) for each configuration are also provided, highlighting the selected configuration for fabrication with the minimum arm length that maintains high force intensity. The chosen configuration for enhanced mechanical stability has an arm length of 180 µm, 12 fingers, and a finger length of 15 µm, with a force intensity of 184.8 N/m^2^.

*L_arm_* [μm]	(*N_f_*, *L_f_*) [1, μm]	*σ Range* $\left[\frac{N}{m^{2}}\right]$
180	(8,22.5), (9,20), (10,18), (11,16.4), (12,15), (13,13.8)	181.0–187.7
190	(8,23.8), (9,21.1), (10,19), (11,17.3), (12,15.8), (13,14.6), (14,13.6)	181.4–191.4
200	(8,25), (9,22.2), (10,20), (11,18.2), (12,16.7), (13,15.4), (14,14.3), (15,13.3)	180.8–193.8
210	(8,26.3), (9,23.3), (10,21), (11,19.1), (12,17.5), (13,16.2), (14,15), (15,14), (16,13.1)	180.5–195.6
220	(9,24.4), (10,22), (11,20), (12,18.3), (13,16.9), (14,15.7), (15,14.7), (16,13.8), (17,12.9), (18,12.2)	181.0–197.7
230	(9,25.6), (10,23), (11,20.9), (12,19.2), (13,17.7), (14,16.4), (15,15.3), (16,14.4), (17,13.5), (18,12.8)	186.0–198.8
240	(9,26.7), (10,24), (11,21.8), (12,20), (13,18.5), (14,17.1), (15,16), (16,15), (17,14.1), (18,13.3), (19,12.6)	185.6–199.9
250	(9,27.8), (10,25), (11,22.7), (12,20.8), (13,19.2), (14,17.9), (15,16.7), (16,15.6), (17,14.7), (18,13.9), (19,13.2), (20,12.5)	184.4–201.3
260	(9,28.9), (10,26), (11,23.6), (12,21.7), (13,20), (14,18.6), (15,17.3), (16,16.3), (17,15.3), (18,14.4), (19,13.7), (20,13)	183.2–203.3
270	(9,30), (10,27), (11,24.5), (12,22.5), (13,20.8), (14,19.3), (15,18), (16,16.9), (17,15.9), (18,15), (19,14.2), (20,13.5)	181.3–203.3
280	(9,31.1), (10,28), (11,25.5), (12,23.3), (13,21.5), (14,20), (15,18.7), (16,17.5), (17,16.5), (18,15.6), (19,14.7), (20,14)	180.5–204.8
290	(10,29), (11,26.4), (12,24.2), (13,22.3), (14,20.7), (15,19.3), (16,18.1), (17,17.1), (18,16.1), (19,15.3), (20,14.5)	184.8–204.4
300	(10,30), (11,27.3), (12,25), (13,23.1), (14,21.4), (15,20), (16,18.8), (17,17.6), (18,16.7), (19,15.8), (20,15)	184.0–206.3
310	(10,31), (11,28.2), (12,25.8), (13,23.8), (14,22.1), (15,20.7), (16,19.4), (17,18.2), (18,17.2), (19,16.3), (20,15.5)	182.7–206.6
320	(10,32), (11,29.1), (12,26.7), (13,24.6), (14,22.9), (15,21.3), (16,20), (17,18.8), (18,17.8), (19,16.8), (20,16)	181.3–207.0
330	(10,33), (11,30), (12,27.5), (13,25.4), (14,23.6), (15,22), (16,20.6), (17,19.4), (18,18.3), (19,17.4), (20,16.5)	181.0–207.1
340	(11,30.9), (12,28.3), (13,26.2), (14,24.3), (15,22.7), (16,21.3), (17,20), (18,18.9), (19,17.9), (20,17)	185.0–207.4
350	(11,31.8), (12,29.2), (13,26.9), (14,25), (15,23.3), (16,21.9), (17,20.6), (18,19.4), (19,18.4), (20,17.5)	183.0–207.4

## Data Availability

The data that support the findings of this study are available from the corresponding author upon reasonable request.
